# Effects of Mixed Fruits and Berries on Ameliorating Gut Microbiota and Hepatic Alterations Induced by Cafeteria Diet

**DOI:** 10.3390/nu18020181

**Published:** 2026-01-06

**Authors:** Rawan Al Hazaimeh, Louis Shackelford, Judith Boateng

**Affiliations:** Department of Food and Animal Sciences, Alabama A&M University, 4900 Meridian Street North, Normal, AL 35762, USA; rawan.alhazaimeh@aamu.edu (R.A.H.);

**Keywords:** adolescent obesity, gut microbiota, polyphenols, gut–liver axis, cafeteria diet

## Abstract

**Background/Objectives:** The study investigated the potential of mixed fruits and berries (MFB) as a dietary intervention to mitigate cafeteria (CAF) diet-induced gut microbiome dysbiosis and hepatic dysfunction associated with metabolic syndrome and steatohepatitis (MASH) in an adolescent rat model. **Methods:** Forty-eight adolescent male Sprague-Dawley rats (*n* = 3 cages per group (two rats per cage)) were divided into eight experimental groups, where NC received the normal AIN-93G basal diet, PC received the CAF diet and normal AIN-93G basal diet, T_1_ and T_2_ received MFB supplementation (3% and 6% levels) without CAF exposure, P_1_ and P_2_ received a MFB (3% and 6% levels) supplementation initiated at the onset of CAF feeding, and I_1_ and I_2_ received MFB supplementation initiated 2 weeks after CAF feeding. After 6 weeks, cecal 16S rRNA, hepatic histopathology, Oil Red O staining, and metabolic dysfunction-associated steatotic liver disease (MASLD)-related biomarkers (liver enzymes, alanine aminotransferase (ALT), and aspartate aminotransferase (AST)) were analyzed. **Results:** AST: ALT ratio was the highest in the PC group (3.63, *p* < 0.05) compared to the MFB groups. Oil Red O staining showed lower hepatic lipid accumulation, and histological analysis demonstrated a marked reduction in portal inflammatory cell infiltration in MFB. Alpha diversity (Simpson Index) decreased in PC (Kruskal–Wallis, *p* = 0.043). CAF increased *Lactobacillus johnsonii* (+75%, *p* < 0.05), while reducing *L. murinus* and *L. intestinalis* (~90%, *p* < 0.05). MFB supplementation restored *Bifidobacterium Pseudolongum* and increased *Akkermansia muciniphila* levels in the P_2_, I_1_, and I_2_ groups (~20-fold, *p* < 0.05). *Bacteroides dorei* was present in all groups except the PC group. These bacteria presented a positive correlation with key SCFAs. **Conclusions:** The results from this study indicated that MFB supplementation modulated gut microbiota composition and enhanced SCFA production, thereby strengthening intestinal barrier integrity and reducing gut-derived inflammation. Collectively, these effects attenuated hepatic lipid accumulation and inflammation, highlighting the potential of MFB to restore gut–liver axis homeostasis disrupted by CAF-induced dysbiosis in adolescent rats.

## 1. Introduction

Emerging evidence highlights the fundamental role of diet in shaping the composition and function of the gut microbiome [1,2,3,4]. Initial colonization of the gut microbiota begins at birth and continues to develop throughout life, with diet playing a crucial role [5], particularly during adolescence when microbial shaping remains highly dynamic. A healthy diet during this critical developmental stage has been shown to prevent stress-induced alterations in gut microbiota [6]. Furthermore, distinct gut bacterial and metabolite profiles correlated with body mass index (BMI) in adolescents, thus highlighting the complex interactions between diet, the microbiome, and metabolic health [7].

Several studies have demonstrated that consuming a Cafeteria-style or Western diet during adolescence induces gut dysbiosis, characterized by a pronounced reduction in microbial α-diversity (richness and evenness) and an increase in β-diversity [4,8]. This dysbiotic shift reduces beneficial bacteria, such as *Bifidobacterium*, and increases pathogenic species, including *Escherichia coli* [9,10]. Furthermore, intestinal dysbiosis, particularly shifts in the Firmicutes-to-Bacteroidetes (F/B) ratio, compromises the intestinal barrier and facilitates the translocation of lipopolysaccharides (LPS) into the systemic circulation, leading to metabolic endotoxemia. This process, along with the accumulation of trimethylamine-*N*-oxide (TMAO) and secondary bile acids, collectively triggers systemic low-grade inflammation. Such chronic inflammation is a key driver in the pathogenesis of Metabolically Dysfunctional-Associated Fatty Liver Disease (MAFLD) [11]. The progression of MAFLD to advanced-stage fibrosis and hepatocellular carcinoma often originates from early-life metabolic disturbances and persistent microbial dysbiosis [12]. Therefore, unhealthy dietary patterns during adolescence can alter the gut microbiome imbalance, lead to a disruption in the gut–liver axis, and potentially predispose adolescents to chronic liver injury and long-term risk of liver fibrosis and cancer [13,14].

One of the primary metabolites produced by gut microbiota is Short-chain fatty acids (SCFAs). SCFAs, primarily acetate, propionate, and butyrate, are produced when gut microbiota ferment dietary fiber and other prebiotics. These metabolites serve as energy substrates to support the growth and maintenance of various tissues, including the intestinal epithelium and hepatocytes, thus promoting gut barrier integrity and liver metabolic health [15,16]. SCFAs also modulate host metabolism and immune responses through several pathways, including the AMP-activated protein kinase (AMPK) pathway, the Peroxisome Proliferator-Activated Receptor gamma (PPAR-γ) pathway, and histone deacetylase inhibition (HDAC), thereby contributing substantially to gut–liver homeostasis [17,18]. An imbalance in gut-derived SCFAs, particularly a reduction in butyrate, resulting from a Cafeteria or Western diet, can compromise gut barrier integrity through the translocation of lipopolysaccharides (LPS) and other microbial metabolites into portal circulation, contributing to systemic inflammation, metabolic endotoxemia, and increased risk of MAFLD [19,20]. Several studies have shown that systemic inflammation can lead to the subsequent activation of hepatic Toll-like receptor 4 (TLR4) signaling and Kupffer cell-mediated inflammatory responses. These actions perturb lipid metabolism and promote hepatic steatosis, thereby linking gut-derived disturbances to liver metabolic dysfunction [21,22,23,24,25].

Studies have shown a strong positive correlation between polyphenol-rich fruits and berries and the gut microbiota. Polyphenols actively stimulate the growth of beneficial bacteria such as *Bifidobacterium* and *Lactobacillus* [26,27]. For example, anthocyanins from blueberries and blackberries beneficially modulate gut microbiota composition by increasing the abundance of *Prevotella histicola*, *Lactobacillus*, and members of the phylum *Bacteroidetes*, while reducing the abundance of *Allobaculum* and *Actinobacteria*. These microbial shifts elevated acetic acid production, which in turn improved several metabolic syndrome parameters [28]. This study provides clear evidence that polyphenols act as prebiotics, promoting the growth of beneficial microbiota and enhancing the production of short-chain fatty acids (SCFAs). Mechanistically, SCFAs produced by polyphenol-stimulated gut microbiota play a key role in the gut–liver axis. Acetate, propionate, and butyrate signal through G-protein–coupled receptors (GPR41, GPR43, GPR109A) on intestinal epithelial and immune cells, modulating inflammation and enhancing the secretion of hormones such as GLP-1 and PYY [29,30]. These effects enhance intestinal barrier integrity, reduce endotoxin translocation, and lower hepatic exposure to gut-derived inflammatory mediators, collectively mitigating liver inflammation and steatosis [31]. In particular, butyrate serves as an energy source for colonocytes and strengthens tight junctions, thereby limiting gut permeability and preventing the activation of hepatic Kupffer cells by microbial products [15]. Thus, polyphenol-induced modulation of the gut microbiota and SCFA production represents a crucial mechanism linking diet, gut health, and liver function through the gut–liver axis.

While fruits and berries may also help to prevent chronic diseases due to their health-improving compounds, intake among adolescents is significantly low. According to the Dietary Guidelines for Americans (ADG), adolescents often fail to meet the recommended daily fruit consumption benchmark of 1.5–2 cups. The failure to meet this recommendation reveals a critical opportunity for early dietary intervention. Given the increasing prevalence of adolescent obesity and its progression to long-term metabolic disorders, targeting the gut–liver axis through polyphenol-rich dietary intervention offers a promising approach. Furthermore, supplementation with single fruits or berries has been well-documented as a means of mitigating obesity-related health issues. However, mechanistic studies on the combination of fruit and berry interventions in a standardized Cafeteria/Western diet model to mimic adolescent obesogenic eating patterns remain scarce. This study aims to fill this gap by connecting dietary strategies to the mechanisms of gut–liver crosstalk, a critical area rarely explored in adolescent obesity. The choice of the mixed fruits and berries (MFB) formulation used in this study represents the fruits and berries that dominate U.S. adolescent intake patterns. The proportional amounts in the blend increase the diversity of polyphenol contents.

Therefore, we aimed to determine the potential role of MFB to mitigate cafeteria (CAF) diet-induced gut microbiome dysbiosis. We hypothesized that MFB polyphenols would attenuate diet-induced MAFLD in adolescents by modulating the gut–liver axis. Specifically, we expect increased SCFA (particularly butyrate), reduced hepatic lipid accumulation, and lower ALT/AST, reflecting improved gut barrier function and decreased inflammatory spillover to the liver. Accordingly, the present study employed a CAF-based model with MFB supplementation initiated in the absence of CAF exposure (T), concurrent with CAF feeding (P), or two weeks following CAF feeding (I).

## 2. Materials and Methods

### 2.1. Diet Preparation and Composition

This study was conducted using the same animal cohort described in our previous publication [32], under the same approved ethical protocol. No data from that publication is duplicated here; the present manuscript analyzes distinct endpoints collected from the same animals. The freeze-dried mixed fruits and berries (MFB) powder, which contained blueberries, blackberries, raspberries, pineapple, strawberries, peaches, and mangos, was prepared as previously reported [32]. The MFB blend was prepared by incorporating it into a standard diet (AIN-93G) at two concentrations: 3% and 6% [32] (Supplementary Table S1) and was designated as the treatment diets. The MFB supplementation represents the equivalent of one and two recommended servings per day of fruits for human adolescents.

**Figure 1:**
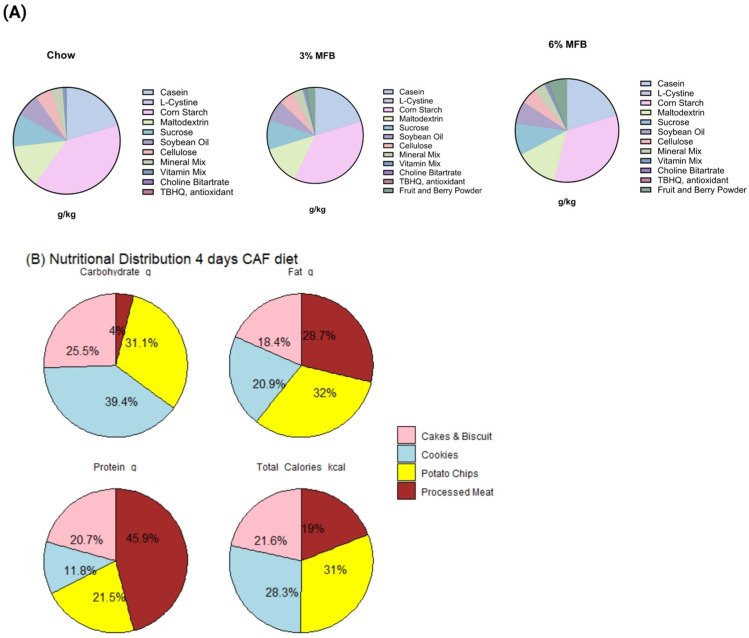
Composition of Treatment and CAF diets (**A**) nutrient content in treatment diets (AIN-93G, 3% MFB, and 6% MFB) (g/kg) and (**B**) macronutrients and total calorie content for the 4-day cycle CAF diet (%).

Both the treatment (MFB-supplemented diets) and control (AIN-93G) diets were formulated by Envigo (Madison, WI, USA) to be isocaloric (Figure 1A) (Supplementary Table S1). The diets were cold-pelleted with Moisture content of 12% and were not irradiated. They were then dried at 50 °C for 4 h, thereby minimizing the loss of heat-sensitive compounds and nutrients, such as phytochemicals, minerals, and vitamins. Pelleted diets were stored in air-tight containers at 4 °C until use. The CAF diet used in this study was based on the protocol of Lalanza and Snoeren [33], with modifications to accommodate U.S. market brands [32]. The CAF diet cycle (4 days) consisted of four choices, two savory and two sweets, designed to induce hyperphagia. The food items were selected from the following categories: cakes, pastries, cookies, ultra-processed meats (including sausages, bacon, and salami), flavored snack chips, sweets/candies, and breads (such as breadsticks). The composition and macronutrient content of the CAF diets are provided in Supplementary Table S2. Ten grams of the food items offered were rotated regularly with new food items. A 10% (*w*/*v*) sucrose solution was provided ad libitum, alongside water, to mimic high-calorie liquid intake [32] (Figure 1). Rats were given ad libitum access to the CAF diet alongside access to standard AIN-93G or experimental diets [34,35]. Food intake was monitored daily by weighing the remaining food in the feeders.

The macronutrient composition of the CAF diet (~51% fat (17% saturated), 6% protein, and 43% carbohydrate (10% sugar)) has been previously reported [32] (Figure 1B). The weekly caloric intakes from the CAF, MFB, or AIN-93G diets (g), and sucrose drink (mL) per cage were converted into kilocalories (kcal) and totaled. The daily calorie intake (kcal) and food efficiency (Feed efficiency (g/kcal) = [Body weight gain (g)/Feed intake (Kcal)] × 100) were calculated as previously described [32].

### 2.2. Animal Housing and Study Design

Sample Size Determination: The sample size per group (*n*) was determined based on prior rodent studies and power calculations performed using SAS, as previously described [32]. Using an alpha level of 0.05 and a statistical power of 0.90, the calculated sample size was six rats per group. An additional 10% was included to account for anticipated attrition or mortality.

**Figure 2:**
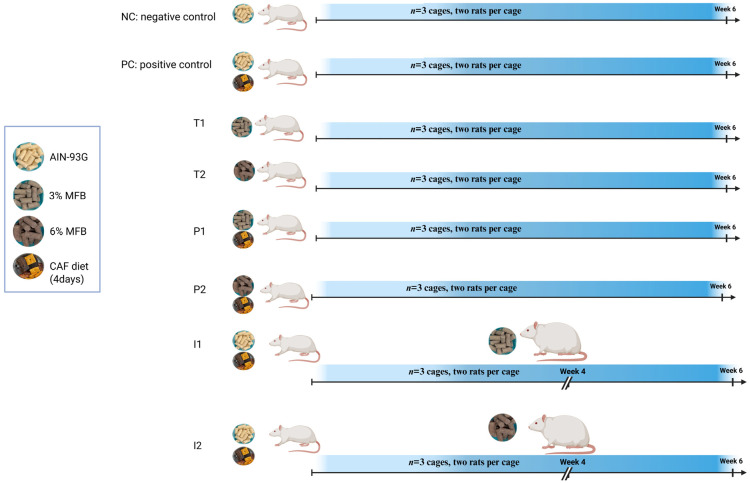
Experimental layout of adolescent Sprague Dawley rats (37PND) into eight different groups (Created in BioRender. Al Hazaimeh, R. (2025) https://BioRender.com/5x4rub9).

Animal Housing: Forty-eight adolescent Sprague Dawley male rats at postnatal day (PND) 30 were obtained from Envigo (Madison, WI, USA). The rats were housed in an environment where the light and dark cycles were maintained at 12 h each, and the temperature and relative humidity were maintained at 21 °C and 50%, respectively. Following a one-week acclimation @ postnatal day 37 (PND-37), Sprague-Dawley rats were divided into eight experimental groups (*n* = 3 cages per group (two rats per cage)) (Figure 2), where NC received the normal AIN-93G basal diet, PC received the CAF diet and normal AIN-93G basal diet, T_1_ and T_2_ received MFB supplementation (3% and 6% levels) without CAF exposure, P_1_ and P_2_ received a MFB (3% and 6% levels) supplementation initiated at the onset of CAF feeding, and I_1_ and I_2_ received MFB supplementation initiated 2 weeks after CAF feeding (Supplementary Table S3) Rats remained on their respective treatments until PND 79.

Ethics Statement: All animal procedures were conducted in accordance with institutional guidelines for the care and use of laboratory animals and were approved by the Institutional Animal Care and Use Committee (IACUC) of Alabama A&M University (Protocol No. 100234, approved on 29 June 2021). Experiments were conducted between 13 September and 28 October 2021.

### 2.3. Plasma Biochemical Measurements

At the end of the 6-week study (PND-79), rats were euthanized via CO_2_ asphyxiation in accordance with institutional animal care and use guidelines. Thoracotomy was performed to expose the heart, and whole blood was collected directly from the left atrium into EDTA-containing tubes. Plasma was obtained by centrifugation (3000× *g* for 15 min at 4 °C) and then stored at −80 °C until analysis. Diagnostic plasma kits for triglycerides (TG), HDL-C, LDL-C, and VLDL-C were purchased from Abcam (Cambridge, MA, USA).

### 2.4. Liver Tissue Preparation for Biochemical Analysis

Liver tissue weighing 100 mg was homogenized in 1 mL of phosphate-buffered saline (PBS) with 10% Ethylenediaminetetraacetic acid (EDTA) using tubes compatible with the Fisherbrand Bead Homogenizer (Fisher Scientific, Suwannee, GA, USA). The homogenized samples were centrifuged at 2000× *g* for 5 min at 4 °C. The resulting supernatant was collected into new microcentrifuge tubes and stored at −80 °C until analysis. The supernatant was used to evaluate hepatic enzymatic activities, including glutathione S-transferase (GST) (nmol/min/mg protein) and glutathione reductase (GR) (nmol/min/mg protein), according to the manufacturer’s instructions (Cayman Chemical Company, Ann Arbor, MI, USA). Liver function markers, hepatic tissue alanine aminotransferase (ALT) (IU/mg protein), and aspartate aminotransferase (AST) (IU/mg protein) were measured according to the manufacturer’s instructions (Cayman Chemical Company, Ann Arbor, MI, USA). The Creatinine level (mg/dL) was measured in the urine using a commercial kit (Cayman Chemical Company, Ann Arbor, MI, USA).

### 2.5. Histopathological Examination of Liver Tissue

For Oil Red O staining, frozen tissue sections were randomly fixed in triplicate in 10% formalin for 10 min at room temperature, rinsed in distilled water, and then briefly immersed in 60% isopropanol to prepare the tissue for staining. The sections were incubated in Oil Red O working solution for 10–15 min, followed by a rinse in 60% isopropanol to remove excess stain. After a final wash in distilled water, nuclei were counterstained with hematoxylin. Sections are then mounted using an aqueous mounting medium, as alcohol-based media can dissolve the lipid-bound dye. Stained tissues were observed under a light microscope (Olympus BX63, Olympus, Tokyo, Japan). Liver samples in triplicate slides, were randomly fixed in 10% neutral-buffered formalin, embedded in paraffin, sectioned at 5 µm, and stained with hematoxylin and eosin (H&E). Histopathological scoring in five randomly selected fields per section was performed according to the criteria described by Takahashi et al. [34], with modifications suitable for CAF diet–induced hepatic steatosis. The evaluation included steatosis, lobular inflammation, portal inflammation, hepatocellular ballooning, necrosis, and fibrosis. Steatosis was graded on a scale of 0–3 based on the percentage of hepatocytes containing lipid droplets (0 = <5%, 1 = 5–33%, 2 = 34–66%, 3 = >66%). Lobular and portal inflammation were scored from 0 to 3 (0 = no foci; 1 = <2 foci per 200× field; 2 = 2–4 foci; 3 = >4 foci). Hepatocellular ballooning was graded 0–2 (0 = none; 1 = few ballooned cells; 2 = many or prominent ballooned cells). Fibrosis was staged separately on a 0–4 scale (0 = none; 1 = perisinusoidal or periportal; 2 = perisinusoidal and portal/periportal; 3 = bridging fibrosis; 4 = cirrhosis). Confluent and focal lytic necrosis were recorded as present (1) or absent (0) to capture early hepatocellular damage patterns. A composite histological profile was then randomly generated for each animal, and mean values per group were calculated for comparative visualization. Lipid droplet quantification was performed using the method of Exner et al. [35], with results expressed as area fractions representing the proportion of lipid-stained regions to the total tissue area.

### 2.6. Short-Chain Fatty Acids (SCFA) Analysis

Short-chain fatty acids (SCFAs) in colonic contents were quantified from six randomly selected biological replicates per group (*n* = 3). Short-chain fatty acids (SCFAs) in cecal contents were quantified following Scheppach et al. [36]. Approximately 100 mg of cecal content was homogenized in 1 mL of deionized water using a Fisherbrand Bead Mill 4 Mini homogenizer (Fisher Scientific, Suwannee, GA, USA) for 2 min. The homogenate was centrifuged at 12,000× *g* for 10 min at 4 °C, and the supernatant was transferred to a clean microcentrifuge tube. Subsequently, 1 mL of ethanol containing 0.5% HCl (*v*/*v*) and 50 µL of acetic acid-d_4_ (internal standard) was added, followed by vortexing and ultrasonic extraction for 40 min. After centrifugation at 14,000 rpm for 10 min, the supernatant was collected for gas chromatography analysis. Quantitative analysis of SCFAs was performed using an Agilent 7890B Gas Chromatograph coupled to an Agilent 5977A Mass Selective Detector (Agilent Technologies, Palo Alto, CA, USA). Separation was achieved on an Agilent DB-FFAP capillary column (30 m × 0.25 mm internal diameter × 0.25 µm film thickness). The injector temperature was maintained at 250 °C, and 1 µL of each sample was injected in splitless mode. The oven temperature program was as follows: an initial temperature of 50 °C was maintained for 2 min, followed by an increase to 120 °C at a rate of 15 °C/min, then to 170 °C at 5 °C/min, and finally to 240 °C at a rate of 15 °C/min, where it was held for 3 min. The carrier gas was high-purity helium at a constant flow rate of 1.0 mL/min. The mass spectrometer was operated in electron ionization (EI) mode at 70 eV, with both full-scan and selected ion monitoring (SIM) acquisition modes. Ion source and quadrupole temperatures were maintained at 230 °C and 150 °C, respectively. Standard solutions of acetic, propionic, isobutyric, butyric, isovaleric, valeric, and hexanoic acids were prepared in ethanol at concentrations ranging from 0.001 to 1000 µg/mL. Calibration curves were generated for each compound by plotting the peak area ratio of the analyte to the internal standard (acetic acid-d_4_) against the corresponding concentration. Linear regression yielded coefficients of determination (R^2^ > 0.99) for all SCFAs. The limit of quantification (LOQ) for each compound was 0.005 µg/mL. SCFA concentrations in samples were calculated from the calibration curves and expressed as µg/g cecal content.

### 2.7. Microbiome DNA Extraction and 16S rRNA Sequencing

At euthanasia, the weights of both the full and empty cecum were recorded, and colon length was measured. The cecum contents were immediately frozen at −80 °C for further analysis. Microbial DNA was extracted from 100 mg of cecum content using the PowerFecal^®^ Pro DNA Kit (Qiagen, Germantown, MD, USA), following the manufacturer’s instructions. Mechanical lysis was performed using bead beating with the FastPrep-24 Instrument (MP Biomedicals, Solon, OH, USA). The concentration and purity of the extracted DNA were assessed using the Qubit™ 4.0 Fluorometer (Thermo Fisher Scientific, Rockville, MD, USA). DNA samples were stored at −80 °C until further analysis. Approximately two μL of the DNA was used to amplify the 16S hypervariable regions using primer V3–V4 hypervariable region of the bacterial 16S rRNA gene using a two-step PCR approach with specific primers: 341F(TCGTCGGCAGCGTCAGATGTGTATAAGAGACAG(spacer)TGCCTACGGGNGGCWGCAG) and 806R (GTCTCGTGGGCTCGGAGATGTGTATAAGAGACAG(spacer)CCGGACTACNVGGGTWTCTAAT). Amplicons were purified and sequenced on an Illumina MiSeq platform (Illumina, Inc., San Diego, CA, USA) using a paired-end 300-cycle run with V3 reagents.

Raw sequences were processed using QIIME2 (version 2022.02), and quality control steps, including demultiplexing, filtering, trimming, and denoising, were performed with the DADA2 plugin. Amplicon sequence variants (ASVs) were taxonomically assigned using a classifier trained on the SILVA 138 database at 99% identity. Taxonomic assignment was conducted at the phylum, class, order, family, and genus levels. The raw 16S rRNA gene sequencing data have been deposited in the NCBI Sequence Read Archive (SRA) under BioProject accession number SUB15752527.

### 2.8. Microbial Diversity and Bioinformatics Analysis

Alpha (α) and beta (β) diversity were computed using QIIME2 (version 2022.02). These metrics were calculated to evaluate microbial diversity within and between samples, respectively. Alpha diversity was assessed at a rarefaction depth of 24,901 reads per sample using the Observed ASV index. Beta diversity was evaluated using UniFrac distance metrics to capture phylogenetic dissimilarities among samples. Principal Coordinate Analysis (PCoA) was applied to visualize patterns of microbial community structure based on the distance matrix. Samples were color-coded by the treatment group to reveal clustering or separation trends in microbial composition.

### 2.9. Statistical and Functional Analysis

Statistical analyses were conducted using SAS software (version 9.4; SAS Institute, Cary, NC, USA). Data were expressed as mean ± standard error of the mean (SEM). A completely randomized design was used to analyze group differences via one-way analysis of variance (ANOVA), followed by Tukey’s post hoc test for pairwise comparisons. The PROC UNIVARIATE procedure was used to assess data normality, and the homogeneity of variances was evaluated to ensure the assumptions of ANOVA were met. Post hoc comparisons were adjusted using Tukey’s test. The average weight gain measure was analyzed using a mixed-effects model, with group, time (in weeks), and the group × time interaction as fixed effects, and individual subjects as a random effect. Differences were considered significant when the *p*-value ≤ 0.05 was obtained (*n* = 3 cages per group, 2 rats per cage). For non-parametric data, the Kruskal–Wallis test was applied to compare groups, followed by pairwise Wilcoxon rank-sum tests. Differential abundance analysis was conducted using linear discriminant analysis effect size (LEfSe), with a significance threshold set at an LDA score greater than 3. Visualization of statistical outputs, including principal component analysis (PCA), heatmaps, and diversity plots were performed using GraphPad Prism (version 5.0) and RStudio (version 4.2.3).

## 3. Results

### 3.1. The Effects of MFB and CAF Diets on Weight and Diet

**Figure 3:**
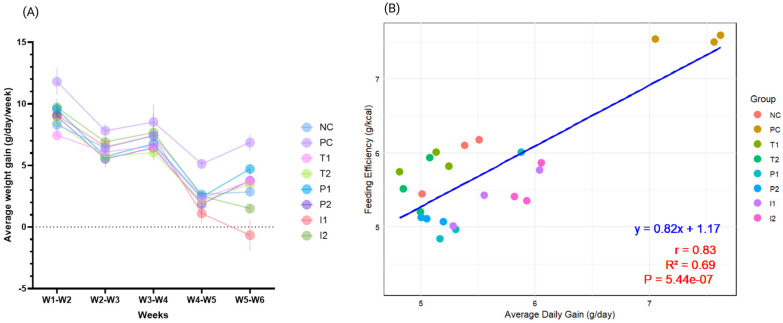
(**A**) Average weekly weight gain (g/day/week) for rats fed different treatments, and (**B**) correlation plot between ADG (g/d) and feeding efficiency (Feed efficiency (g/kcal) = [Body weight gain (g)/Feed intake (Kcal)] × 100) (*n* = 3 cages per group (2 rats per cage) in Sprague-Dawley male rats fed different treatments). Data are presented as mean ± SEM.

The results of the different diets on average weekly weight gain across weeks (W1–W6) are shown in Figure 3A. We noted clear differences in trends (g/day/week) based on dietary interventions. The PC consistently recorded the highest values (*p* < 0.05) throughout the study, compared to NC, T_1_, and T_2_ groups, which showed a progressive decline over time, with lower values observed at later time points. Although the P_1_ and P_2_ displayed moderately higher weight gains compared to the NC, T_1_, and T_2_ groups, particularly during W1–W2 (9.57 ± 0.86 and 9.00 ± 0.30 g/day/week, respectively), a decline was noted thereafter to levels similar to those of the other groups. Interestingly, I_1_ and I_2_ suppressed weight gain most strongly, with I_1_ showing negative growth (−0.67 ± 1.26 g/day/week) and I_2_ a modest increase (1.49 ± 0.13 g/day/week) by W5-W6. Overall, the I_1_ and I_2_ groups attenuated weight gain to levels comparable to those in the NC group, while PC rats maintained significantly elevated growth throughout.

The scatter plot in Figure 3B illustrates the relationship between Average Daily Gain (ADG) (g/day) and Feeding Efficiency (g/kcal) across treatment groups. A linear regression line fitted to the data provided insight into the overall trend. The correlation coefficient (r) quantifies the strength and direction of this relationship, while the R-squared value indicates the proportion of variance in Feeding Efficiency (g/kcal) explained by ADG in grams per day. We observed a positive correlation between ADG and feeding efficiency (r = 0.83, *p* = 5.44 × 10^−7^). This correlation suggests a moderate association between these variables. Overall, the results suggest that the PC group demonstrated the highest feeding efficiency, as reflected by the highest ADG, indicating that the CAF diet is a high-calorie source that efficiently induces significant weight gain. The other groups (P_1_, P_2_, I_1_, and I_2_) clustered with the NC, T_1_, and T_2_ groups.

### 3.2. The Effects of MFB and CAF Diets on Liver Enzymes and Biochemical Analysis

**Figure 4:**
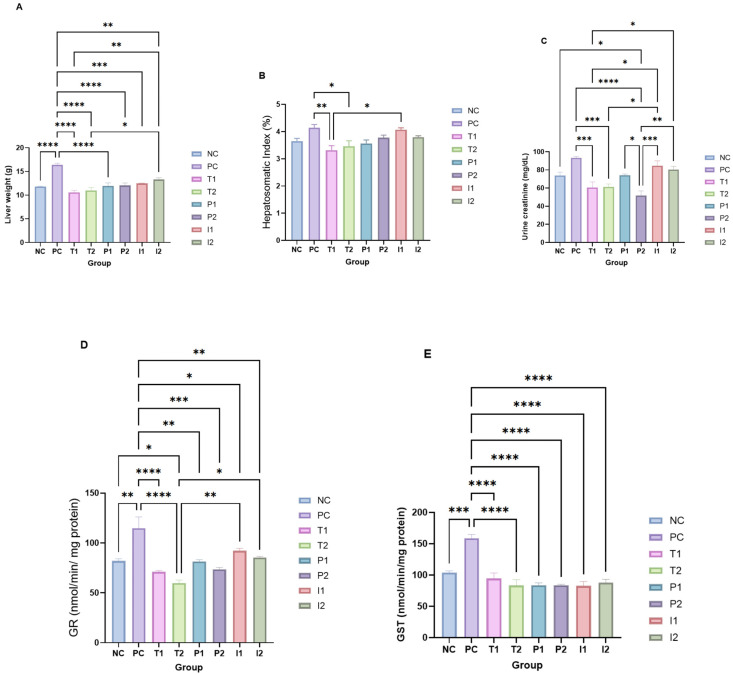
Comparative analysis hepatic study for rats fed different treatments (**A**) Liver weight (g), (**B**) Hepatosomatic index (HSI) ((liver weight (g)/body weight (g) × 100) (%)), (**C**) Urine creatinine (mg/dL), (**D**) GR (nmol/min/mg protein), (**E**) GST (nmol/min/mg protein). (*, **, ***, ****, shows significance at *p*  <  0.05, *p* < 0.01, *p*  <  0.001, *p*  <  0.0001) (*n* = 3 cages per group (2 rats per cage)). Abbreviations: GST—Glutathione S-Transferase, GR—Glutathione Reductase.

Hepatic (liver) weight (g) was measured to reflect the effect of the CAF diet on hepatocytes (Figure 4A). The results indicated that the T_1_ and T_2_ groups had presented the lowest hepatic weights (−32%, *p* < 0.05) compared to the PC. The P_1_, P_2_, I_1_, and I_2_ groups showed a 20% decrease (*p* < 0.05) compared to the PC group, while maintaining comparable hepatic weights to the NC, T_1_, and T_2_ groups. Evaluation of the hepatosomatic index (HSI) (%) (liver weight (g)/body weight (g) × 100) (Figure 4B) suggested that the PC group exhibited the highest ratio (+30%, *p* < 0.05), achieving statistical significance only when compared to the T_1_ and T_2_ groups.

Creatinine level is crucial in evaluating the glomerular filtration rate (GFR) and, consequently, the kidney’s ability to effectively eliminate metabolic waste products from the circulation (Figure 4C). In the present study, urine creatinine levels (mg/dL) were significantly elevated in the PC group compared to the NC group (+31.4%, *p* < 0.05), indicating increased renal output or muscle metabolism. T_1_ and T_2_ showed reduced creatinine levels relative to PC and aligned more closely with the NC group. Similarly, the P_1_ and P_2_ displayed moderate creatinine levels, suggesting partial normalization. In contrast, I_1_ and I_2_ showed variable, but lower levels than PC, with I_2_ presenting the most notable decrease.

The activity of the hepatic glutathione antioxidant system is assessed through the activity of GR (nmol/min/mg protein) (Figure 4D), and GST (nmol/min/mg protein) (Figure 4E). The GST enzyme activity was significantly higher in the PC than NC (+55.5%, *p* < 0.05). P_1_, P_2_, I_1_, and I_2_ exhibited lower activity levels (−46%, *p* < 0.05) compared to the PC group. Likewise, GR activity was significantly higher in PC than in NC (+34%, *p* < 0.05) and the other groups. While the MEB groups’ activities decreased by 27% (*p* < 0.05) compared to the PC.

**Figure 5:**
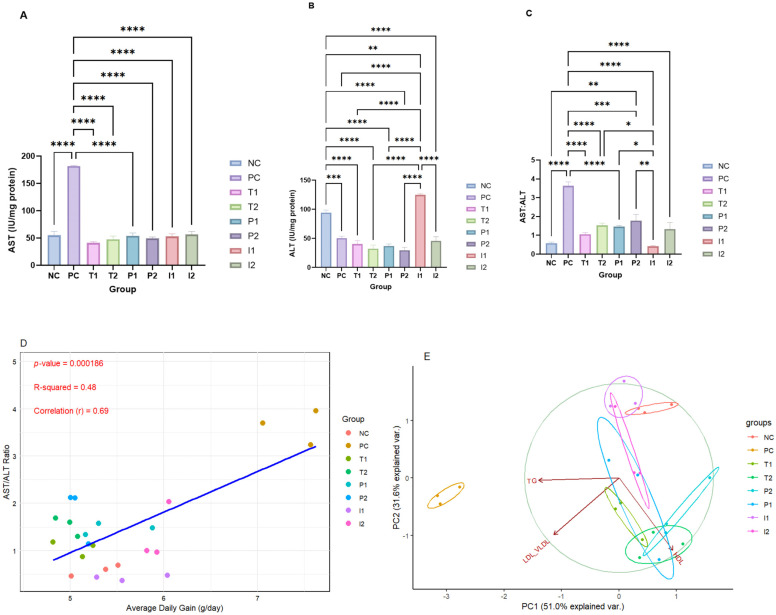
Comparative analysis hepatic study for rats fed different treatments (**A**) AST (IU/mg protein), (**B**) ALT (IU/mg protein), (**C**) AST: ALT ratio, (**D**) Correlation scattered plot of ALT/AST to AGD (g/day), (**E**) PCA lipid profile. (*, **, ***, **** shows significance at *p*  <  0.05, *p* < 0.01, *p*  <  0.001, *p*  <  0.0001) (*n* = 3 cages per group (2 rats per cage)). Abbreviations: AST—Aspartate Aminotransferase, ALT—Alanine Aminotransferase, AST: ALT—Ratio of Aspartate Aminotransferase to Alanine Aminotransferase, PCA—Principal Component Analysis, H&E—Hematoxylin and Eosin (staining).

On the subject of hepatic tissue enzymes AST (IU/mg protein), ALT (IU/mg protein), and their ratio (Figure 5A, B, and C, respectively), AST levels in PC group increased by approximately 228% while ALT levels decreased by approximately 55%. In hepatic tissue, the AST: ALT ratio exhibited variability, with the highest ratio in PC (3.63, *p* < 0.05) and the lowest in the T_1_ group (0.58). In the I_2_ group, there were significant alterations in enzyme levels compared to the NC group. Notably, the AST: ALT ratio in the liver increased by approximately 129% in the I_2_ group compared to the NC group. P_1_ and P_2_ exhibited comparable hepatic AST levels to those of the NC group.

Correlation analysis was conducted between ADG (g/day) and the AST/ALT ratio across treatment groups. The scatter plot (Figure 5D) revealed a moderate positive correlation (r = 0.69, R^2^ = 0.48, *p* = 0.000186), suggesting that higher ADG was generally associated with higher AST/ALT ratios. Notably, the PC group exhibited the highest correlation, whereas P_1_, P_2_, I_1_, and I_2_ clustered more closely with NC, T_1_, and T_2_, indicating similar hepatic enzyme profiles. This trend suggests that increased weight gain may be associated with elevated hepatic enzyme activity, potentially indicating early hepatic stress in the PC group. Moreover, it illustrated the ability of MFB to mitigate the deleterious effects of the CAF diet on hepatic tissue.

PCA of the lipid profile (HDL (mg/dL), LDL/VLDL (mg/dL), and TG (mg/dL) is presented in Figure 5E. Here, PC1 explained 51% and PC2 explained 31.6% of the total variance. Arrows pointing in the same direction indicate a positive correlation, as seen between LDL/VLDL (mg/dL) and TG (mg/dL). In contrast, arrows pointing in the opposite direction represent a negative correlation, such as between HDL (mg/dL) and LDL/VLDL (mg/dL) or TG (mg/dL). The scores plot revealed a distinct separation of the PC group, characterized by hyperlipidemia and hypertriglyceridemia, reflecting the effects of the CAF diet. The P_1_, P_2_, and NC, T_1_, and T_2_ clustered to the right, with a trend toward higher HDL (mg/dL). On the other hand, the I_1_, and I_2_ groups cluster toward the top, correlating with higher LDL/VLDL (mg/dL) and TG (mg/dL) levels.

### 3.3. The Effects of MFB and CAF Diets on Histopathological Study

**Figure 6:**
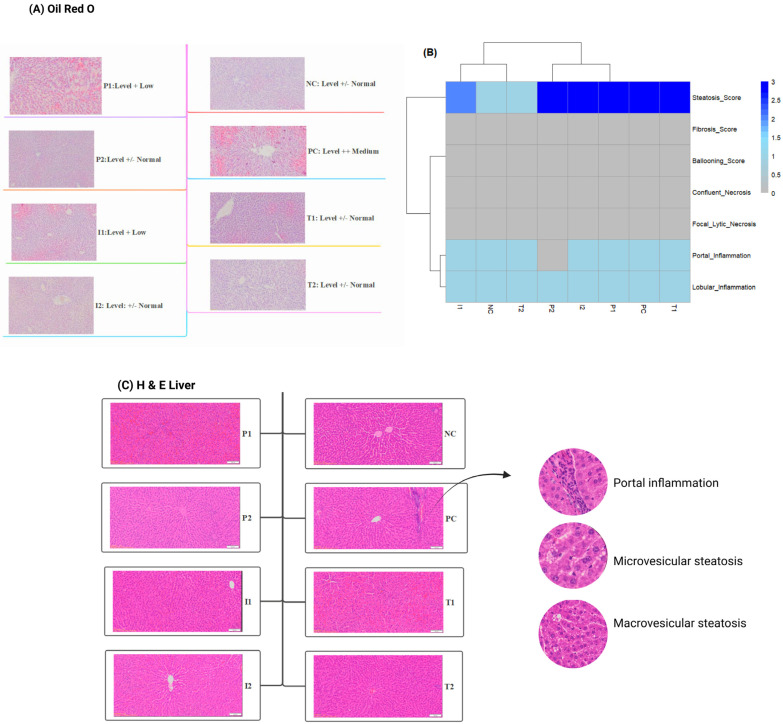
Comparative analysis hepatic study for rats fed different treatments (**A**) Hepatic Oil Red O staining, (**B**) Liver histology score heatmap, and (**C**) Hepatic histological study (H&E staining, 100×). *n* = 3 cages per group (2 rats per cage). Abbreviations: H&E—Hematoxylin and Eosin (staining).

Oil Red O staining revealed critical insights into lipid accumulation within hepatocytes, as shown in Figure 6A. PC group exhibited obvious medium-level lipid accumulation. In contrast, both groups, P_1_ and I_1_, demonstrated markedly reduced hepatocellular lipid accumulation. Remarkably, P_2_ and I_2_ groups displayed the lowest hepatocellular lipid levels, comparable to NC, T_2_, and T_1_ groups.

Histological examination of hepatic tissues and the nonalcoholic steatohepatitis (NASH) activity score were determined (Figure 6B,C). In PC group, approximately 80% of hepatocytes exhibited macrovesicular steatosis, while P_1_, P_2_, T_1_, and I_2_ groups showed approximately 60% of hepatocytes exhibiting macrovesicular steatosis. However, I_1_ and NC groups displayed 30% and 10% of hepatocytes, respectively, while T_2_ showed similar results to NC. There was no necrosis or fibrosis in the liver parenchyma, and none of the hepatocytes exhibited ballooning degeneration in any of the groups. In PC group, most portal areas exhibited mild chronic inflammation, characterized by the presence of inflammatory cells, primarily composed of lymphocytes and macrophages. In contrast, NC, T_1_, and T_2_ groups showed no inflammation in the portal areas. P_1_, P_2_, I_1_, and I_2_ showed mild chronic inflammation in some portal areas. Fibrosis was present in all groups except P_2_.

### 3.4. The Effects of MFB and CAF Diets on Cecum Weight, Colon Length, and SCFA

**Figure 7:**
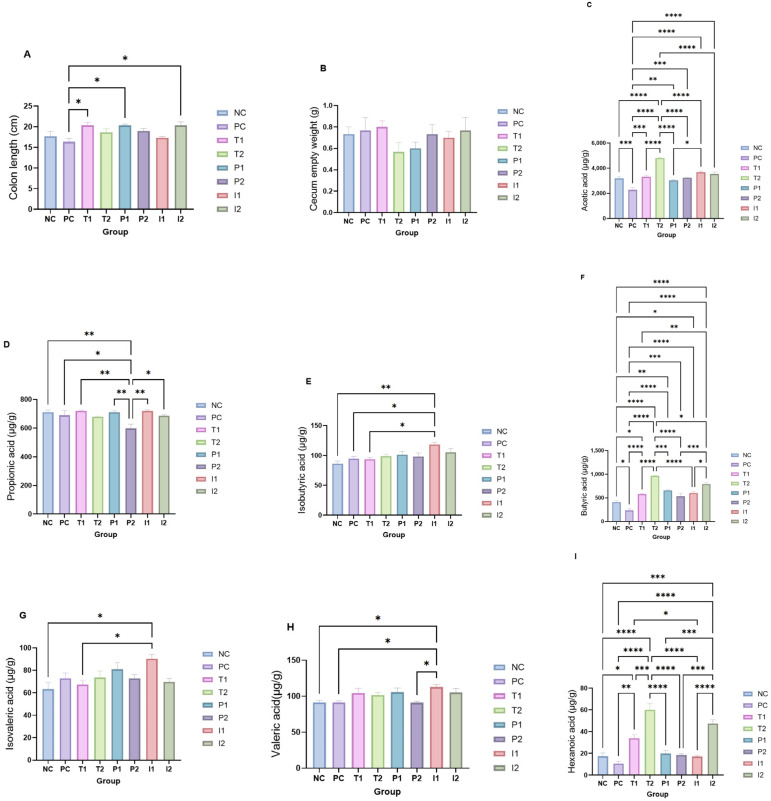
Comparative analysis of rats fed different treatments showing effects on intestinal morphology and cecal fermentation. Panels depict (**A**) colon length (cm), (**B**) empty cecum weight (g), and cecal concentrations (µg/g) of short-chain fatty acids (SCFAs): (**C**) acetic acid, (**D**) propionic acid, (**E**) isobutyric acid, (**F**) butyric acid, (**G**) isovaleric acid, (**H**) valeric acid, and (**I**) hexanoic acid. Data are presented as mean ± SEM. (*, **, ***, **** shows significance at *p* < 0.05, *p* < 0.01, *p* < 0.001, *p* < 0.0001) (*n* = 3 cages per group (2 rats per cage)).

Colon length and cecum weights are presented in Figure 7A and Figure 5B, respectively. As shown, PC group had the shortest colon length (16.3 ± 0.88 cm) of all groups, whereas T_1_, P_1_, and I_2_ (+22.3% compared to PC) were the longest (*p* < 0.05). Meanwhile, NC, T_2_, P_2_, and I_1_ (+10.2% than PC) had colon lengths that were intermediate. Cecum weight ranged from 0.4 to 1 g. The results indicated no significant differences in cecum weight between groups; T_2_ and P_1_ presented the lowest weights (*p*> 0.05).

Figure 7C–I shows SCFAs cecum levels identified in the experimental groups. Acetic acid was the most abundant SCFA, whereas hexanoic acid was the lowest across all groups. According to the results, PC group displayed significantly the lowest acetic acid level (2607.5 ± 176.5 μg/g) among all groups (*p* < 0.05), whereas T_2_ (+84.2% vs. PC), T_1_ (+30%), and I_1_ (+35%) exhibited the highest levels. NC (+22%), P_2_ (+23%), P_1_ (+16%), and I_2_ (+34%) displayed intermediate levels of acetic acid (*p* < 0.05) (Figure 7C). Propionic acid was similar across all groups, showing no significant difference (*p* ˃ 0.05) (Figure 7D). PC group had 734.5 ± 45.42 μg/g, NC (+0.8%), and T_1_ (−3.3%) were close to PC, while T2 (−7%), P_1_ (−5%), P2 (−25%), I_1_ (−3%), and I_2_ (−8%) showed modest reductions. Isobutyric acid concentration was significantly higher in I_1_ (+14.3%) (*p* < 0.05), but lower in NC (−6.4%), T_1_ (−8.7%), T_2_ (−5.3%), P_1_ (−9.1%), P_2_ (−10.9%), and I_2_ (−5.2%) compared with PC (101.1± 3.79 μg/g) (*p* ˃ 0.05) (Figure 7E). Butyric acid was significantly lower in PC at 234.4 ± 39.3 μg/g (*p* < 0.05). T_2_ (+300%), P_1_ (+150%), and I_2_ (+190%) had the highest levels, while T_1_ (+120%) and NC (+54%) had intermediate levels (Figure 7F). Isovaleric acid concentrations were similar across the majority of rat groups, with a PC of 74.3 ± 4.18 μg/g. I_1_ (+11.8%) was the highest, while T_1_ (−13.8%) and T_2_ (−14.3%) were the lowest (Figure 7G).

Overall, valeric acid showed no significant difference between groups, with only I_1_ (+31.6%) (*p* < 0.05) and T_2_ (+15.8%) being higher than PC (90.6 ± 2.38 μg/g), while P_2_ (−2.4%) and NC (+6.9%) were intermediate. Hexanoic acid showed the largest relative variation, with PC being the lowest, at 14.7 ± 3.61 μg/g; however, this difference was only significant when compared with I_2_ and T_1_ and T_2_ groups (*p* < 0.05). T_2_ (+240.8%), T_1_ (+108.5%), and I_2_ (+201.0%) had the highest levels, NC (+48.2%) and P_2_ (+26.1%) were intermediate, and P_1_ (+3.8%) and I_1_ (+9.7%) were close to the PC (Figure 7I).

### 3.5. The Effects of MFB and CAF Diets on the Taxonomy of Cecal Microbiota in the Rat Groups

**Figure 8:**
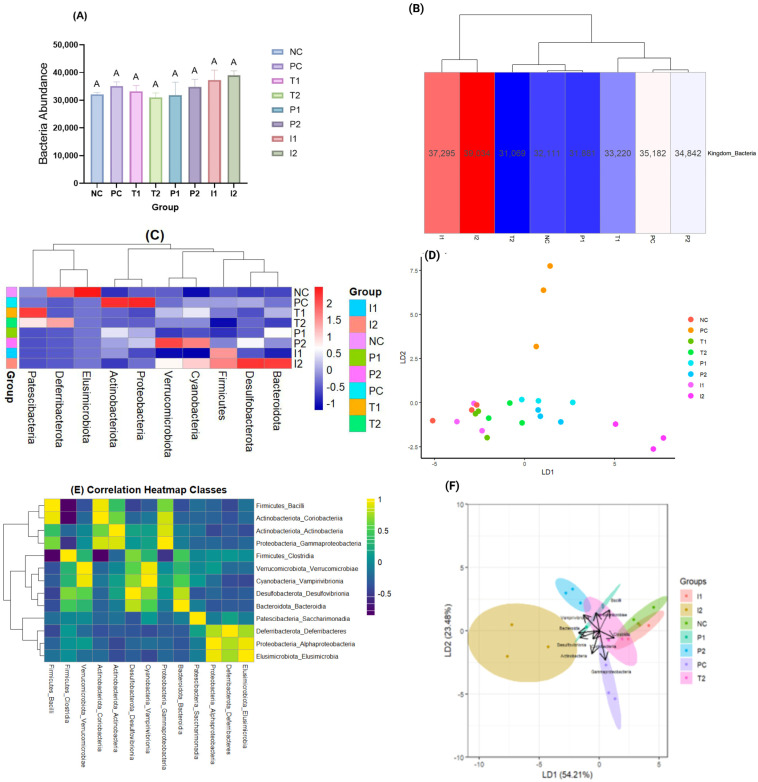
Comparative analysis of microbiota study for rats fed different treatments (**A**) Bacteria Abundance, (**B**) Heatmap for bacteria, (**C**) Heatmap normalized of bacteria phylum abundance, (**D**) Phylum LDA, (**E**) Classes Heatmap, and (**F**) Classes LDA. Data presented as mean ± SEM of Sprague-Dawley rats; different letters between treatments indicate a significant difference (*n* = 3 cages per group (2 rats per cage, values pooled per cage)).

We analyzed 16S rRNA gene sequences to assess the species abundance and composition of cecal microbiota at various taxonomic levels, including Kingdom, Phylum, Class, Order, Family, Genus, and Species. In the bacterial kingdom abundance analysis (Figure 8A), PC group had a mean bacterial abundance of 35,182 cells. I_2_ (+10.9% vs. PC) and I_1_ (+5.9%) exhibited the highest abundances, while T_2_ (−11.7%) and P_1_ (−9.4%) displayed the lowest.

T_1_ (−5.7%), NC (−8.8%), and P_2_ (−1%) showed intermediate values. Overall, no significant differences were found between the groups. The heatmap of bacterial abundance revealed a strong resemblance between the I_1_ and I_2_ groups (Figure 8B), as indicated by their close clustering and comparable abundance profiles. Likewise, P_1_ exhibits clustering with NC, T_2_ groups, whereas PC shows a relationship with P_2_ at the level of abundance.

We identified ten bacterial phyla in this study: Firmicutes, Verrucomicrobiota, Actinobacteriota, Desulfobacterota, Cyanobacteria, Proteobacteria, Bacteroidota, Patescibacteria, Deferribacterota, and Elusimicrobiota (Figure 8C). NC group harbored all ten phyla, whereas the PC group exhibited reduced diversity, consisting of only eight. Specifically, Deferribacterota and Elusimicrobiota were absent in the PC group. The loss of these phyla, although typically present at lower abundance, may indicate a disruption of microbial diversity and potential weakening of community stability, which is often linked to compromised gut resilience and health. In T_1_ and T_2_, eight and nine phyla were populated, respectively. P_1_ and P_2_ each consisted of seven phyla, with Patescibacteria, Deferribacterota, and Elusimicrobiota, compared to NC group. Interestingly, I_1_ exhibited the least diversity with only six phyla identified. According to the data, Firmicutes was the dominant phylum across all groups, accounting for 87.75–93.81% of the total phylum abundance.

The Linear Discriminant Analysis (LDA) results indicate clear differentiation among the bacterial phyla across the different experimental groups, based on LD1 and LD2 (*p* = 4.188^−12^) (Figure 8D). The scatter plot illustrates group clustering, indicating that variations in bacterial composition significantly contribute to the separation of groups. The distinct separation of PC group from all groups highlights the strong impact of the CAF diet on gut microbiota composition at the phylum level. Groups I_2_ and P_2_ are separated along LD1, suggesting differences in their microbial profiles. Clustering of P_1_ and I_1_ with T_1_, T_2_, and NC groups indicated partial restoration. Overall, Firmicutes, Verrucomicrobiota, and Actinobacteriota made a significant contribution to the observed differences, as their abundance varied across the groups.

Figure 8E represents the correlation heatmap between bacteria classes. Firmicutes, Bacilli, and Clostridia were the most abundant classes overall and were particularly dominant in NC, PC, and I_1_ groups. Notably, Bacilli were markedly elevated in PC and P_1_ groups. Likewise, the Verrucomicrobiota phylum, *Verrucomicrobiae* class, showed higher relative abundance in P_2_ and I_2_ groups. The Actinobacteria classes were moderately abundant across all groups, with higher values observed in the PC group. Less abundant classes, such as *Gammaproteobacteria*, *Saccharimonadia*, and *Vampirivibrionia*, displayed group-specific increases, especially in T1, T2, P_1_, and P_2_ groups. NC group had relatively balanced microbial class distributions, serving as a reference for natural microbial diversity. The Linear Discriminant Analysis (LDA) conducted at the class level scaled microbial abundance data across experimental groups (*p* = 0.007) (Figure 8F). Notably, I_1_ and I_2_ clustered closely, suggesting similar microbial patterns, whereas distinct separation was observed for PC, indicating differing microbial community structures. Key contributing taxa to the group separation included *Clostridia*, *Bacilli*, and *Coriobacteriia*, as they showed considerable variance across groups. Analysis revealed that the CAF diet was associated with increased relative abundance of *Bacilli* and *Clostridia*.

**Figure 9:**
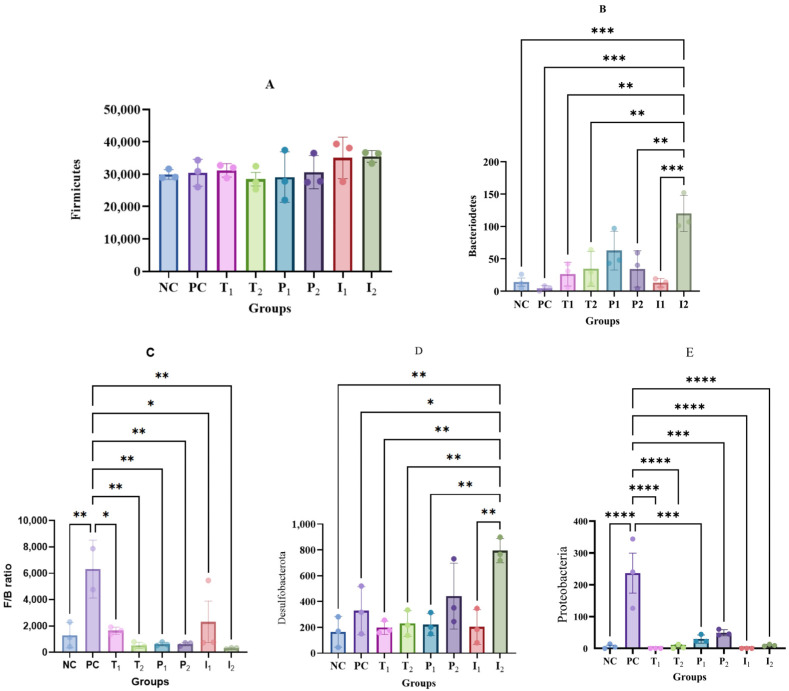
Comparative analysis of the phylum for rats fed different treatments. (**A**) Firmicutes, (**B**) Bacteroidetes, (**C**) Firmicutes and Bacteroidetes ratio (F/B ratio), (**D**) Desulfobacterota, and (**E**) Proteobacteria. Data are presented as mean ± SEM. (*, **, ***, **** shows significance at *p*  <  0.05, *p* < 0.01, *p*  <  0.001, *p*  <  0.0001) (*n* = 3 cages per group (2 rats per cage, values pooled per cage)).

Phyla abundance is presented in Figure 9. Firmicutes (Figure 9A) dominated across all groups, with 80–95% of the total phyla and no significant difference between groups. Bacteroidota accounted for less than 1% of the total phyla (Figure 9B), with the highest abundance observed in I_2_ (0.3%) and the lowest in PC group, with less than 0.01%. CAF diet resulted in a 69% reduction in Bacteroidetes in PC group compared to NC group, and a 78% reduction when compared to the I_2_ group. However, P_2_ and I_1_ showed results comparable to NC. While Figure 9C presents the Firmicutes-to-Bacteroidetes (F/B) ratio, this widely accepted key indicator reflects the balance of gut microbiota. The F/B ratio was highest in PC group (6317.62), with T_2_ and I_2_ showing the lowest ratios.

Desulfobacterota (Figure 9D) and Proteobacteria (Figure 9E) showed no significant differences. Deferribacterota was significantly higher in I_2_ compared to all groups (*p* < 0.05), except for P_2_. Proteobacteria were substantially more abundant in PC group (263.67) (*p* < 0.05), with no observable growth in T_1_ and I_1_. The lowest levels were observed in PC group (Figure 7D, *p* < 0.05).

### 3.6. Richness and Diversity of Cecal Microbiota

**Figure 10:**
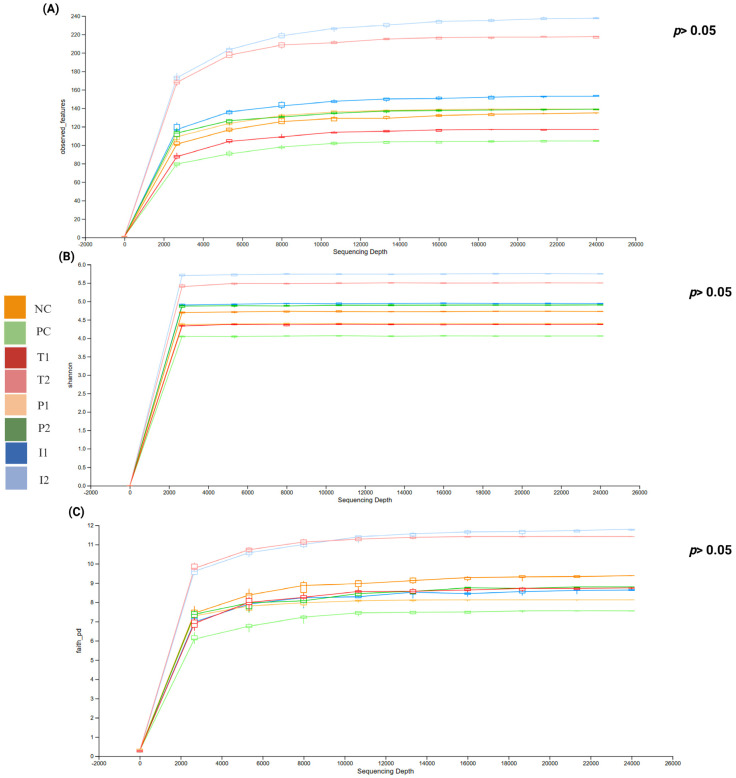

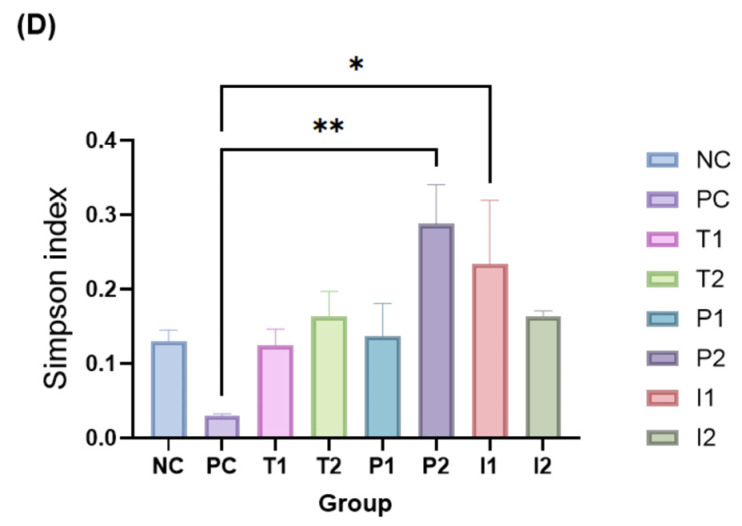
Microbiota alpha and beta diversity for rats fed different treatments. Alpha diversity: (**A**) Observed feature, (**B**) Shannon, (**C**) Phylogenetic Diversity, and (**D**) Simpson diversity index. Plots were visualized using QIIME View. Data are presented as mean ± SEM. (*, ** shows significance at *p* < 0.05, *p* < 0.01) (*n* = 3 cages per group (2 rats per cage, values pooled per cage)).

We assessed the alpha diversity of cecal microbiota at the genus level using Observed Features (ASV richness), Shannon Index (richness and evenness), Faith’s Phylogenetic Diversity (evolutionary relationships), and Simpson Index (evenness and dominance) (Figure 10A–D). QIIME View box plots represent the distribution of each metric at even sampling depth; the lower and upper whiskers correspond to the 9th and 91st percentiles, and the box edges represent the 25th and 75th percentiles. Overall, no statistically significant differences were noted in Observed Features, Shannon Index, or Faith’s Phylogenetic Diversity across groups (*p* > 0.05). PC group exhibited the lowest values for these metrics, suggesting reduced richness and diversity, whereas I_2_ and T_2_ groups showed the highest values. P_1_ and P_2_ maintained alpha diversity levels comparable to NC group. The Simpson Index showed a statistically significant difference among groups (Kruskal–Wallis, *p* = 0.043), with higher index values indicating greater diversity. Additionally, rarefaction curves plateaued for all samples, suggesting that sequencing depth was sufficient to capture the microbial diversity present. A clear separation in alpha diversity profiles was observed between I_1_, I_2_, P_1_, and P_2_ groups and PC group.

**Figure 11:**
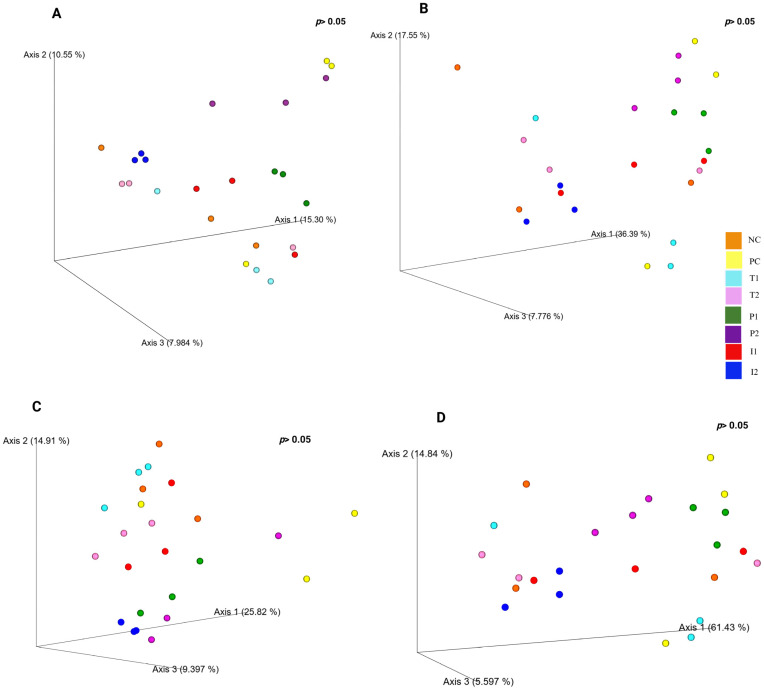
Microbiota d beta diversity for rats fed different treatments. Emperor beta diversity: (**A**) Jaccard Index, (**B**) Bray–Curtis, (**C**) Unweighted, and (**D**) Weighted UniFrac metrics for microbial community comparison, at the ASV level. Plots were visualized using QIIME View. (*p* < 0.05; *n* = 3 cages per group) (2 rats per cage, values pooled per cage).

Beta diversity, assessed through Jaccard Index, Bray–Curtis, Unweighted, and weighted phylogenetic clustering and principal coordinates analysis (PCoA) (Figure 11A–D, respectively). QIIME View showed that the first three components (PC1, PC2, and PC3) of the total variance, respectively. Jaccard Index indicating an overlap in species presence between rat groups, basically ignoring abundances. Overall, P_1,_ I_1_, and I_2_ groups showed some degree of similarity with NC, T_1_, and T_2_ groups in their gut microbiota structure, with overlapping clusters, whereas P_2_ increased microbial diversity, distributed in different directions.

### 3.7. Comparative Analysis of Obesity-Associated Microbiota Species

In this study, we identified a total of 212 microbial species. Table 1 highlights a selection of highly represented species within each phylum. Firmicutes presented the majority, with Family *Lactobacillaceae*, Genus Lactobacillus (*Lactobacillus johnsonii, Lactobacillus murinus, Lactobacillus intestinalis, Lactobacillus reuteri*). Family *Streptococcaceae* also appeared prominently, featuring *Lactococcus lactis* (Genus *Lactococcus*) and multiple *Streptococcus* taxa, including *Streptococcus agalactiae*. Beyond these, we observed other notable Firmicutes families such as *Lachnospiraceae*, which included genera like *Blautia*, *Lachnoclostridium*, and *Roseburia*, as well as Ruminococcaceae, represented by Ruminococcus and *Incertae Sedis*. Outside the Firmicutes, the phylum Actinobacteriota included recognized genera such as *Bifidobacterium* (*Bifidobacterium pseudolongum, Bifidobacterium animalis,* and *Rothia)*, while Verrucomicrobiota was represented by *Akkermansia muciniphila*. We also identified members of Bacteroidota, including *Bacteroides dorei*, and *Proteobacteria*, including the genus *Escherichia Shigella*.

Within the *Lactobacillaceae* family, *Lactobacillus* was the dominant genus, with *Lactobacillus johnsonii* exhibiting the greatest abundance among all taxa. CAF-fed groups (P_1_, P_2_, and I_1_) showed significantly higher levels than NC, T_1_, and T_2_ groups, with PC group exhibiting the greatest increase, nearly fourfold above NC, T_1_, and T_2_ groups (*p* < 0.05; *n* = 3 cages per group, two rats per cage). In contrast, I_2_ displayed abundances comparable to those of the NC, T_1_, and T_2_ groups. *Lactobacillus murinus* was most enriched in NC, T_1_, T_2_, P_2_, I_1_, and I_2_ groups, reaching ~18-fold higher than PC. Conversely, CAF diet reduced *Lactobacillus intestinalis* by 185-fold in PC relative to NC, T_1_, and T_2_ groups (*p* < 0.05), whereas MFB supplementation mitigated this reduction despite CAF intake. Although no significant differences were observed among groups in the abundance of *Lactobacillus* *reuteri*, its levels tended to be higher in CAF-fed rats (*p* > 0.05). In contrast, the genus *Lactococcus*, particularly *Lactococcus lactis*, was most enriched in I_1_, I_2_, P_1_, and P_2_ groups, with significantly higher levels than in PC group, which showed a 37% reduction relative to NC, T_1_, and T_2_ groups (*p* < 0.05). Uncultured *Clostridiales* appeared exclusively in CAF-fed groups, although their abundance was lower in I_1_, I_2_, P_1_, and P_2_ groups, which did not differ significantly from the PC group.

*Bifidobacterium Pseudolongum*, an acetate-producing probiotic, was the lowest in PC group, while T_2_ (6% MFB) was the only group that showed significant inhibition compared with I_1_, I_2_, P_1_, and P_2_ groups, and PC (*p* < 0.05). The intestinal symbiont *Akkermansia muciniphila* was significantly enriched in P_2_, I_1_, and I_2_, whereas its abundance was lowest in PC group. The Gram-negative anaerobe *Bacteroides dorei* was detected in all groups except PC (*p* < 0.05). *Escherichia-Shigella*, a pathogenic group comprising *Escherichia* and *Shigella*, was identified only in PC and I_2_, with a marked proliferation in PC, where its abundance was 27-fold higher than in I_2_ (*p* < 0.05).

### 3.8. Correlation of SCFA-Producing Bacterial Abundance with Acetic and Butyric Acid Levels

**Figure 12:**
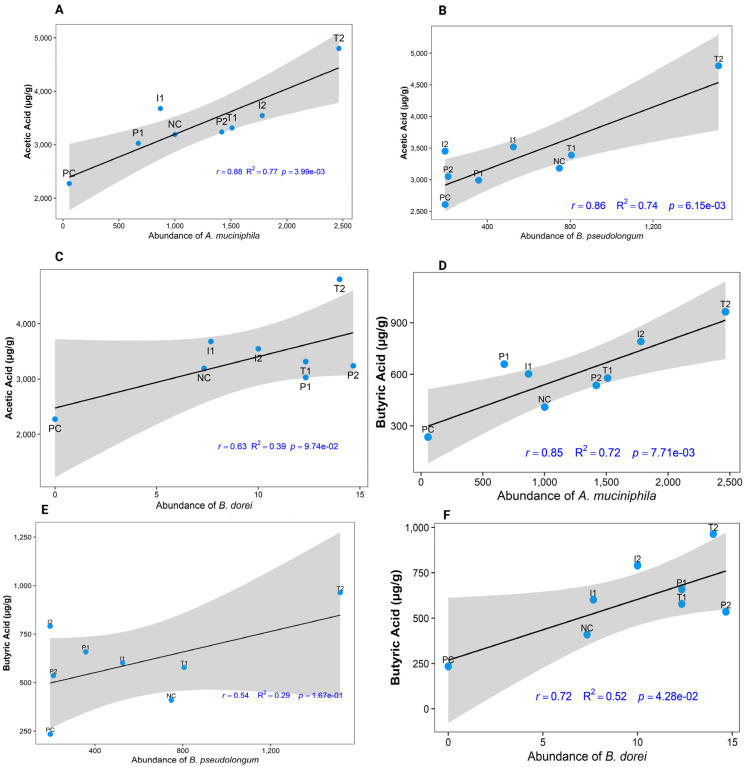
Correlation between acetic acid and butyric acid concentrations and the relative abundance of selected SCFA-producing bacterial species. (**A**) acetic acid (µg/g) with abundance of *Akkermansia muciniphila,* (**B**) acetic acid (µg/g) with abundance of *Bifidobacterium pseudolongum,* (**C**) acetic acid (µg/g) with abundance of *Bacteroides dorei*, (**D**) butyric acid (µg/g) with abundance of *Akkermansia muciniphila,* (**E**) butyric acid (µg/g) with abundance of *Bifidobacterium pseudolongum*, and (**F**) butyric acid (µg/g) with abundance of *Bacteroides dorei.* Only correlations with *p* < 0.05 were considered significant (*n* = 3 cages per group, 2 rats per cage, values pooled per cage).

*Bifidobacterium pseudolongum*, *Akkermansia muciniphila*, and *Bacteroides dorei* are well-recognized bacteria that produce SCFAs and contribute to gut barrier function and host metabolic regulation. Figure 12 illustrates the correlations between acetic acid and butyric acid concentrations and the relative abundance of these SCFA-producing bacterial species. Overall, T_2_ exhibited the strongest positive correlations between cecal acetic and butyric acid levels and the abundance of the three bacterial species, whereas the PC group showed the weakest correlations (*p* < 0.05). Group P_2_ displayed significantly higher butyric acid levels, which were positively correlated with the abundance of *A. muciniphila* and *B. dorei* (Figure 12D,F). In contrast, acetic acid levels in *B. dorei* showed clustering of P_2_ with P_1_ and T_1_ groups (Figure 12C). Moderate acetic acid concentrations were observed to cluster between I_1_ and NC groups in relation to *A. muciniphila* and *B. dorei abundance* (Figure 12A,C).

## 4. Discussion

The present study demonstrated that MFB supplementation effectively mitigates the adverse metabolic and hepatic effects of a CAF/Western diet in adolescent rats. Importantly, the observed biochemical and histological improvements were complementary rather than contradictory, indicating that both systemic and tissue-level protection occurred simultaneously. MFB reshaped the gut microbiota, which showed a strong correlation with improved hepatic function, highlighting the pivotal role of the gut–liver axis.

Although both the current and previous studies utilized the same rat cohort [32], the focus of the present manuscript is distinct. The previous publication primarily addressed obesity-related outcomes, including body weight gain, ADG, feed efficiency, adiposity, and systemic inflammatory markers, whereas the current work investigates gut microbiota composition, short-chain fatty acid production, and gut–liver axis signaling, providing mechanistic insights that were not reported previously.

As previously reported, MFB improved the obesogenic effects induced by the CAF diet in terms of body weight gain [32], average daily gain (ADG), and feeding efficiency. Further findings from the present study demonstrated significant weight reductions within just two weeks of MFB supplementation, highlighting its rapid impact, which is likely mediated through the modulation of metabolic processes rather than the direct regulation of gene expression. In this context, the present findings suggest that MFB supplementation may influence metabolic outcomes by modulating gut dysbiosis and enhancing antioxidant defenses, potentially mediated by its fiber and polyphenol-rich composition [32,37,38,39,40]. These effects may occur through the regulation of adipogenesis, enhancement of antioxidant defenses, and inhibition of digestive enzymes, which are attributable to the bioactive properties of MFB [32,37,38,39,40].

The CAF diet is recognized for inducing rapid weight gain, adiposity, and hepatic dysfunction, with the liver being particularly vulnerable to fat accumulation, steatosis, and metabolic dysregulation [41,42,43,44,45,46,47]. In the present study, we found that PC group (fed predominantly with CAF diet) exhibited increased liver weight, fat deposition, marked steatosis, and an elevated hepatosomatic index (HSI). In contrast, MFB supplementation preserved hepatic morphology, lowered lipid accumulation, and alleviated pathological alterations. Although histological improvements were evident, these changes should be interpreted in conjunction with biochemical markers rather than as evidence of direct molecular mechanisms. It is likely that these hepatoprotective effects may be partly explained by MFB’s enriched polyphenol content, which supports redox balance and modulates liver enzyme activity.

The glutathione reductase system is crucial for maintaining redox balance and protecting cells from damage induced by prooxidants [48]. In this study, CAF-fed rats exhibited significantly elevated GST and GR activities, indicating an adaptive enzymatic response to heightened oxidative stress. This is consistent with reports that CAF diets upregulate GST enzymes [49], elevate oxidative and inflammatory markers in the gastrointestinal tract and adipose tissue [32,38], and increase proinflammatory cytokines such as IL-6 and TNF-α [41,47]. By contrast, I_1_, I_2_, P_1_, and P_2_ groups supplemented with MFB exhibited reduced GST and GR activities, suggesting a lower oxidative burden. The consistency between reduced enzymatic stress markers and preserved hepatic histoarchitecture strengthens the interpretation that MFB exerted a protective, rather than curative, effect against oxidative injury. These findings align with evidence that antioxidant-rich diets restore redox balance, attenuate inflammation, and improve glutathione homeostasis by increasing glutathione levels and enhancing the GSH: GSSG ratio [48,49]. The upregulation of the glutathione system may reflect an early pathogenesis mechanism of hepatic steatosis. MFB’s ability to restore redox homeostasis may therefore play a critical role in preventing liver injury by preventing lipid deposition and oxidation.

Liver enzymes AST, ALT, and the AST/ALT ratio are biomarkers of hepatic injury, and their elevation reflects steatosis and liver metabolic dysfunction [30,50]. Here, the CAF-fed PC group exhibited elevated enzyme levels, an AST/ALT ratio greater than 1, and histological features of MAFLD, including micro- and macrovesicular steatosis, as well as fibrosis. These outcomes are consistent with reports linking high-fat, high-sugar diets to hepatic lipid accumulation [51,52] and with evidence that excess fructose, saturated fat, and inadequate fiber intake contribute to the pathogenesis of MAFLD [53]. For example, aberrant glucose metabolism has been reported to increase the risk of MAFLD [54]. In particular, insulin resistance disrupts hepatic lipid homeostasis by enhancing *de novo* lipogenesis, reducing β-oxidation, and promoting lipid accumulation, along with pro-inflammatory signaling within hepatocytes [55]. Our study found that MFB supplementation normalized enzyme levels, reduced steatosis, and preserved hepatic architecture in I_1_, I_2_, P_1_, and P_2_ groups, with stronger effects observed at 6% compared to 3%. Furthermore, Oil Red-O staining confirmed a dose-dependent reduction in hepatic lipid storage. Taken together, these benefits are likely mediated by MFB bioactive compounds, primarily anthocyanins, flavonoids, and fiber, which synergistically restored metabolic balance by activating AMPK, suppressing de novo lipogenesis, enhancing β-oxidation [56], and supporting gut microbiota and SCFA production [57]. Collectively, these findings highlight MFB as a promising dietary strategy for preventing and managing CAF-induced MAFLD.

Beyond their role as energy substrates, SCFAs regulate host metabolism, immunity, and gut–liver interactions, making them central to the pathogenesis of obesity and MAFLD. This study found that, butyrate, a key fuel for colonocytes, was lowest in PC group, indicating the CAF diet-induced suppression of its production. The Western diet, rich in fat and poor in fiber, disrupts the colon-SCFA endotoxemia axis by reducing microbial fermentation and depleting butyrate-producing taxa such as *Megasphaera elsdenii* [58].

A recent study found that prolonged exposure to a Western diet in mice resulted in a decrease in colon length [48], which is consistent with findings in PC group. Shortened colon length is associated with a reduction in the production of SCFAs [59], particularly butyrate, which is critical for maintaining epithelial integrity and anti-inflammatory signaling. These alterations impair gut barrier integrity, promote Lipopolysaccharide (LPS) leakage into circulation, and trigger metabolic endotoxemia, thereby exacerbating systemic inflammation, insulin resistance, obesity, MAFLD progression, and tumorigenesis [60,61]. MFB supplementation modulated the gut microbiota by enhancing the production of acetic, butyric, and hexanoic acids. Our study reported that P_2_ showed the highest butyric acid levels, while I_1_ and I_2_ groups exhibited elevated acetic acid levels, correlating with the abundance of the acetic acid-producing probiotic *Bifidobacterium pseudolongum*. These results are consistent with previous studies, which have shown that polyphenol-rich interventions promote butyrogenic taxa, such as *Faecalibacterium prausnitzii* and *Roseburia* [62,63]. Recent studies further support the notion that fiber and polyphenols from berries enhance gut barrier integrity by enriching butyrate-producing bacteria and reducing endotoxemia [64,65].

Analysis of alpha diversity, which reflects the richness of microbial communities, revealed that both I_1_, I_2_, P_1_, and P_2_ groups, which received MFB, exhibited the highest diversity indices. In contrast, PC group showed a significant reduction in diversity. These results align with previous studies that have demonstrated the adverse effects of high-fat, high-sugar diets on gut microbiota diversity [66,67]. Firmicutes and Bacteroidetes dominate the gut microbiota, with other phyla adding complexity [68]. High-fat diets increase the Firmicutes/Bacteroidetes (F/B) ratio, which is associated with disruptions in microbial balance and loss of diversity [69,70,71]. We found that MFB supplementation restored microbial balance and diversity, normalizing the F/B ratio. These shifts corresponded with elevated acetic and butyric acid in I_2_ and P_2_, highlighting MFB’s potential to support gut and metabolic health. Similarly, contemporary research has demonstrated that fiber and polyphenol-rich fruits, particularly those rich in anthocyanins, can counteract CAF-induced dysbiosis by reducing the F/B ratio and restoring microbial diversity [72,73].

Probiotic-rich foods in the CAF diet, such as cheese-containing crackers and breadsticks, may have contributed to the abundance of *Lactobacillus* observed in I_1_, I_2_, P_1_, and P_2_ groups, consistent with previous reports [74]. These probiotics, in conjunction with the prebiotics in MFB, likely supported the growth and activity of *Lactobacillus*, thereby enhancing the beneficial effects on the gut microbiota. I_1_, I_2_, P_1_, and P_2_ groups maintained *Lactobacillus murinus* and were enriched in acetate-producing *Bifidobacterium pseudolongum*, likely through the consumption of prebiotic fibers and polyphenols. In contrast, CAF-fed PC showed elevated Proteobacteria, particularly *Escherichia-Shigella* (*p* < 0.05), reflecting obesity-associated gut dysbiosis [75,76]. These microbiota shifts mirror human MAFLD and non-alcoholic steatohepatitis, characterized by altered *Lactobacillus*, *Bacteroidetes*, and *Firmicutes* profiles, underscoring the potential of dietary prebiotics to modulate gut microbes and mitigate metabolic liver disease [77,78].

*Akkermansia muciniphila*, a Gram-negative bacterium from the phylum Verrucomicrobia, is strongly correlated to metabolic health. It colonizes the gut mucus layer, supports mucosal integrity, and modulates inflammation; its abundance inversely correlates with the prevalence of obesity and type 2 diabetes [79,80]. Large-scale human data, including over 10,000 participants in the American Gut Project, confirm that higher levels of *A. muciniphila* are associated with a reduced risk of obesity, independent of confounders [81]. We noted that P_2_ group fed 6% MFB had the highest abundance of *A. muciniphila*, followed by I_1_ (3% MFB post-obesity induction), while the CAF-fed PC group showed the lowest. This suggests a protective role against obesity-induced inflammation. *A. muciniphila* also produces SCFAs, such as propionate and butyrate, which enhance gut barrier function; correspondingly, P_2_ showed elevated butyrate levels. Moreover, *Bacteroides dorei* has several beneficial effects on the host, including modulating the gut microbiota and potentially reducing the risk of cardiovascular disease by lowering cholesterol. This is achieved mechanistically by boosting interferon production, downregulating inflammation, altering the gut microbiome, and influencing bile acid metabolism [82]. Our results presented the proliferation of this bacterium in all groups except PC.

While a definitive microbial signature for obesity remains elusive, our study provides valuable insights into how dietary interventions, such as MFB, can modulate the gut microbiota to counteract the deleterious effects of a CAF diet. By enhancing microbial diversity and promoting the growth of beneficial bacteria, such as *Akkermansia muciniphila*, *Bacteroides dorei*, and *Lactobacillus*, MFB treatment may help restore microbial equilibrium and alleviate obesity-related inflammation. These findings underscore the complex relationship between gut microbiota composition and metabolic health, highlighting the potential of interventions such as MFB to support overall well-being and prevent or mitigate obesity. Consistent with previous studies, our study found that diet was a dominant factor in shaping gut microbial communities. After a detailed analysis, we identified specific taxa responsible for the differences observed between dietary groups and systematically evaluated the effects of MFB on mitigating the deleterious impact of the CAF diet on gut microbiota, revealing that the administration of MFB led to significant improvements in gut microbiota composition, particularly when compared to the detrimental effects of a CAF diet.

## 5. Conclusions

This study provides initial observations suggesting that dietary supplementation with mixed fruits and berries (MFB) may ameliorate obesogenic outcomes associated with cafeteria (CAF) feeding, including features consistent with metabolic dysfunction-associated fatty liver disease (MAFLD). MFB supplementation was associated with favorable changes in hepatic outcomes, gut microbiota diversity, and indicators of metabolic balance, supporting its potential role in alleviating diet-induced metabolic disturbances during adolescence. Although this investigation represents a short-term study, future work incorporating longer intervention durations beyond six weeks, together with targeted assessment of circulating and fecal polyphenol-derived metabolites, will be essential to strengthen mechanistic understanding and support future clinical evaluation of MFB-based dietary strategies.

## Figures and Tables

**Table 1:** Comparative analysis abundance of cecum microbiota taxa in Sprague-Dawley rats fed different treatments.

Taxonomy	NC	PC	T_1_	T_2_	P_1_	P_2_	I_1_	I_2_
**Phylum/Species (%)**								
***Firmicutes***								
**Genus** *Lactobacillus*								
*Lactobacillus johnsonii*	3064 ± 456 ^D^	12,139 ±508 ^A^	2711 ± 472 ^D^	2734 ±1085 ^D^	10,382 ± 810 ^AB^	6565 ± 404 ^C^	8643 ± 552 ^BC^	2050 ± 423 ^D^
*Lactobacillus murinus*	999 ± 352 ^A^	55.3 ± 14.8 ^C^	801 ± 87.2 ^AB^	847 ± 175 ^AB^	133 ± 65 ^BC^	571 ± 128 ^ABC^	651 ± 227 ^ABC^	774 ± 8.54 ^ABC^
*Lactobacillus intestinalis*	740 ± 198 ^AB^	3.67 ± 2.03 ^C^	761 ± 48.6 ^AB^	814 ± 218 ^A^	1287 ± 218 ^A^	780 ± 73.7 ^A^	159 ± 22.4 ^BC^	1310 ± 102 ^A^
*Lactobacillus reuteri*	269 ± 53.8 ^A^	842 ± 200 ^A^	342 ± 125 ^A^	320 ± 155 ^A^	812 ± 166 ^A^	762 ± 171 ^A^	608 ± 297 ^A^	729 ± 194 ^A^
**Genus** *Lactococcus*								
*Lactococcus lactis*	531 ± 86 ^A^	198 ± 65.7 ^B^	697 ± 253 ^A^	692 ± 142 ^A^	305 ± 18 ^A^	394 ± 69.5 ^A^	738 ± 260 ^A^	915 ± 69.2 ^A^
**Genus** *Clostridia*								
*Uncultured Clostridiales*	0 ± 0 ^B^	11 ± 4.16 ^A^	0 ± 0 ^B^	0 ± 0 ^B^	7.67 ± 1.76 ^AB^	1.67 ± 1.67 ^AB^	2 ± 2 ^AB^	2.33 ± 2.33 ^AB^
**Actinobacteriota**								
*Bifidobacterium Pseudolongum*	748 ± 395 ^AB^	196 ± 106 ^B^	806 ± 145 ^AB^	1517 ± 48.1 ^A^	358 ± 175 ^B^	211 ± 65.3 ^B^	526.36 ± 207 ^B^	196 ± 97.2 ^B^
**Verrucomicrobiota**								
*Akkermansia muciniphila*	1001 ± 44.6 ^BC^	56.7 ± 21.9 ^D^	1510 ± 84.4 ^BC^	2464 ± 119 ^A^	674 ± 278 ^CD^	1419 ± 224 ^BC^	870 ± 48.2 ^BCD^	1780 ± 352 ^AB^
**Bacteroidota**								
*Bacteroides dorei*	7.33 ± 1.20 ^AB^	0 ± 0 ^B^	12.3 ± 2.60 ^A^	14 ± 3.79 ^A^	12.3 ± 3.93 ^A^	14.7 ± 1.45 ^A^	7.67 ± 1.45 ^AB^	10 ± 2.89 ^AB^
**Proteobacteria**								
**Genera** *Escherichia -Shigella*	0 ± 0 ^B^	184 ± 53.6 ^A^	0 ± 0 ^B^	0 ± 0 ^B^	0 ± 0 ^B^	0 ± 0 ^B^	0 ± 0 ^B^	6.67 ± 3.76 ^B^

Data presented as mean ± SEM of Sprague-Dawley rats; different letters between treatments indicate a significant difference (*p* < 0.05; *n* = 3 cages per group (2 rats per cage, values pooled per cage)).

## Data Availability

The data supporting the study’s findings are included in the article. More details are available on request.

## Supplementary Materials

The following supporting information can be downloaded at: https://www.mdpi.com/article/10.3390/nu18020181/s1, Table S1: Diet Composition of AIN-93G, 3% MFB, and 6%MFB; Table S2. Nutrient Composition of the Cafeteria Diet (CAF); Table S3. Experimental diet composition, and timing of MFB supplementation across study groups.

## Abbreviations

ADG—Average daily gain; AIN-93G—American Institute of Nutrition-93 Growth (diet formulation); ANOVA—Analysis of Variance, ALT—Alanine Aminotransferase; AST—Aspartate Aminotransferase; CAF—Cafeteria (diet); DW—Dry Weight; EDTA—Ethylenediaminetetraacetic Acid; GR—Glutathione Reductase; GST—Glutathione S-Transferase; HDL-c—High-Density Lipoprotein Cholesterol; H&E—Hematoxylin and Eosin; LDL-c—Low-Density Lipoprotein Cholesterol; MAFLD—Metabolic-Associated Fatty Liver Disease; MFB—Mixed Fruits and Berries; MAFLD—Metabolic Dysfunction-Associated Fatty Liver Disease; PBS—Phosphate-Buffered Saline; PND—Postnatal Day; SCFAs—Short-Chain Fatty Acids; VLDL-c—Very Low-Density Lipoprotein Cholesterol. OTUs—Operational taxonomic units.
