# Supplementary material for: Effects of Mixed Fruits and Berries on Ameliorating Gut Microbiota and Hepatic Alterations Induced by Cafeteria Diet
Source: Nutrients. 2026 Jan 6;18(2):181. doi: 10.3390/nu18020181 (PMC12845326; doi:10.3390/nu18020181)
Supplement: Supplementary file 1 [file nutrients-18-00181-s001.zip › Al Hazaimeh et al. Table S1.pdf]

Supplementary Table S1. Diet Composition of AIN-93G, 3% MFB, and 6%MFB

Nutrient	Fruit & Berry Powder Diet (3%, 93G)	Fruit & Berry Powder Diet (6%, 93G)	AIN-93G Purified Diet (TD.94045)
**Protein, % by weight**	17.8	18.0	17.7
**Carbohydrate, % by weight**	59.9	59.8	60.1
**Fat, % by weight**	7.3	7.3	7.2
**Protein, % kcal from**	19.0	19.1	18.8
**Carbohydrate, % kcal from**	63.7	63.4	63.9
**Fat, % kcal from**	17.4	17.5	17.2
**kcal/g**	3.8	3.8	3.8

Ingredient	Fruit & Berry Powder Diet (3%, 93G)	Fruit & Berry Powder Diet (6%, 93G)	TD.94045 (AIN-93G)
Casein	200.0	200.0	200.0
L-Cystine	3.0	3.0	3.0
Corn Starch	367.486	337.486	397.486
Maltodextrin	132.0	132.0	132.0
Sucrose	100.0	100.0	100.0
Soybean Oil	70.0	70.0	70.0
Cellulose	50.0	50.0	50.0
Mineral Mix (AIN-93G-MX, 94046)	35.0	35.0	35.0
Vitamin Mix (AIN-93-VX, 94047)	10.0	10.0	10.0
Choline Bitartrate	2.5	2.5	2.5
TBHQ (antioxidant)	0.014	0.014	0.014
**Fruit and Berry Powder**	**30.0**	**60.0**	—

Diets with isocaloric daily intake were measured per cage.
