# Supplementary material for: Effects of Mixed Fruits and Berries on Ameliorating Gut Microbiota and Hepatic Alterations Induced by Cafeteria Diet
Source: Nutrients. 2026 Jan 6;18(2):181. doi: 10.3390/nu18020181 (PMC12845326; doi:10.3390/nu18020181)
Supplement: Supplementary file 1 [file nutrients-18-00181-s001.zip › Al Hazaimeh et al. Table S2.pdf]

Supplementary Table S2. Nutrient Composition of the Cafeteria Diet (CAF)

Menu	Category	Product	Brand	Total Calories (kcal)	Fat (saturated) grams	Carbohydrate (sugar) grams	Protein (grams)	Fiber (grams)	Sodium (mg)
1	Cakes & Biscuit	Muffins	Chocolate Chip Muffins (Walmart)	374.2	18.2 (5.1)	47.5 (29.3)	5.1	2	0
	Cookies	Cream Cookies	OREO Chocolate Sandwich Cookies	491	20.6 (5.6)	73.5 (11.8)	2.9	3	0
	Processed Meat	Hot dog	Ball Park Bun Size Beef Hot Dogs	329.9	28.3 (11.3)	7.5 (1.9)	11.3	0	905.7
	Potato Chips	Classic Chips	Pringles Original	537.2	31.6 (10.5)	57.9 (0)	5.3	5.3	12631.6
	**Total**			**1732.3**	**98.7 (32.5)**	**186.4 (43)**	**24.6**	**10.3**	**13537.3**
2	Cakes & Biscuit	Pastries	Marketside Chocolate Croissants	383.3	21.6 (10)	45 (16.7)	6.7	3.3	0
	Processed Meat	Salami	HORMEL NATURAL CHOICE Sliced Uncured Hard Salami Lunch Meat	406.9	35.7 (14.3)	0 (0)	21.4	0	1607.1
	Cookies	Chocolate Cookie	CHIPS AHOY! Chewy Chocolate Chip Cookies	458.2	19.4 (9.7)	67.7 (35.5)	3.2	3.2	7741.9
	Bread	Flavored breadsticks	New York Bakery Original Bread Sticks with Real Garlic	382	14 (4)	54 (6)	10	2	4800
	**Total**			**1630.4**	**90.7 (38)**	**166.7 (58.2)**	**41.3**	**8.5**	**14149**
3	Cakes & Biscuit	Doughnuts	Freshness Guaranteed Powdered Sugar Mini Donuts (Walmart)	387.8	15.8 (7)	56.1 (24.6)	5.3	3.5	491.2
	Processed Meat	Bacon	Great Value Thick Cut Bacon Hickory Smoked Fully Cooked (Walmart)	555.2	44.4 (13.9)	5.6 (5.6)	33.3	0	2055.6
	Candies	Gumdrop	Tootsie Dots Assorted Fruit Flavored Gumdrops Candy	327.2	0 (0)	81.8 (50)	0	0	0
	Potato Chips	Cheese corn chips	Doritos Tangy Cheese Tortilla Chip	513.3	28.5 (3.6)	57.1 (3.6)	7.1	3.6	8571.4
	**Total**			**1783.5**	**88.7 (24.5)**	**200.6 (83.8)**	**45.7**	**7.1**	**11118.2**
4	Cakes & Biscuit	Brownie	Just a bite brownies - Ultimate Chocolate Brownies	470	24 (6)	54.5 (33)	6	0	90
	Processed meat	Hot dogs	Oscar Mayer Classic Uncured Wieners Hot Dogs	280	22 (6.7)	2 (2)	10	0	420
	Candies	Marshmallow	Jet-Puffed Mini Marshmallows	333	0 (0)	83 (56.7)	0	0	25
	Breads	Flavor cracker	Better Cheddars Baked Snack Cheese Crackers	520	27.6 (3)	58.6 (0)	10	0	828
	**Total**			**1603**	**73.6 (15.7)**	**198 (91.7)**	**26**	**0**	**1363**

*** Standard chow and water are always available for the rodents in the CAF diet group. Values expressed in kcal and (g) per 100g of the product.**
