# Supplementary material for: Effects of Mixed Fruits and Berries on Ameliorating Gut Microbiota and Hepatic Alterations Induced by Cafeteria Diet
Source: Nutrients. 2026 Jan 6;18(2):181. doi: 10.3390/nu18020181 (PMC12845326; doi:10.3390/nu18020181)
Supplement: Supplementary file 1 [file nutrients-18-00181-s001.zip › Al Hazaimeh et al. Table S3.pdf]

Table S3. Experimental diet composition, and timing of MFB supplementation across study groups

Experimental Groups	Base Diet	CAF Diet	MFB Supplementation	Timing of MFB Initiation
NC	AIN-93G	No	No	None
PC	AIN-93G	Yes	No	None
T_1	AIN-93G	No	Yes	From study onset
T_2	AIN-93G	No	Yes	From study onset
P_1	AIN-93G	Yes	Yes	Initiated at onset of CAF feeding
P_2	AIN-93G	Yes	Yes	Initiated at onset of CAF feeding
I_1	AIN-93G	Yes	Yes	Initiated 2 weeks after CAF feeding
I_2	AIN-93G	Yes	Yes	Initiated 2 weeks after CAF feeding

NC; normal control group receiving the basal AIN-93G diet
PC; positive control group receiving the cafeteria-style (CAF) diet
T_1 and T_2; treatment control groups receiving MFB supplementation without CAF exposure
P_1 and P_2; early MFB supplementation groups in which supplementation was initiated at the onset of CAF feeding
I_1 and I_2, delayed MFB supplementation groups in which supplementation was initiated 2 weeks after the start of CAF feeding.
CAF- cafeteria-style diet; MFB- mixed fruits and berries
